# Background commentary on the Researching the Obesogenic Food Environment (ROFE) project

**DOI:** 10.1017/S136898002300280X

**Published:** 2024-01-17

**Authors:** Reginald Adjetey Annan, Nana Ama Frimpomaa Agyapong, Anne Marie Thow, Elizabeth Catherina Swart

**Affiliations:** 1 Department of Biochemistry and Biotechnology, Kwame Nkrumah University of Science and Technology, Kumasi, Ghana; 2 Department of Clinical Nutrition and Dietetics, College of Health and Allied Sciences, University of Cape Coast, Cape Coast, Ghana; 3 Menzies Centre for Health Policy, University of Sydney, Sydney, NSW 2006, Australia; 4 Department of Dietetics and Nutrition, University of the Western Cape, Bellville, South Africa

**Keywords:** ROFE project, South Africa, Ghana, Food environment

## Abstract

**Objective:**

The objective of this commentary is to provide an overview of the rationale and objectives of the Researching the Obesogenic Food Environment (ROFE) project that was conducted in Ghana and South Africa.

**Design:**

Narration has been used to describe the main objectives, phases as well as the methods used for the conduct of this project.

**Setting:**

The project described in this commentary was conducted in Khayelitsha and Mount Frere in South Africa and Ahodwo and Ejuratia for Ghana.

**Participant:**

Participants of the study described here include households in South Africa and Ghana, stakeholders and policymakers, and various actors within the food chain in both countries.

**Results:**

The ROFE findings provide a good understanding of the extent of the impact of the food environment on consumption, characteristics of value chains of healthy and unhealthy foods, as well as the potential for improved governance and policy that is relevant to the region. The supplement provides the opportunity to share the extensive findings of the ROFE project. Nine papers that describe the process and findings of the three phases of the ROFE project have been presented. Some of the papers focus on phases of the ROFE, while others cut across different phases and explore the linkages between the phases. Briefly descriptions of key findings of some of the papers in the supplement are provided.

**Conclusion:**

Together, the findings of the ROFE study presented in this supplement have increased understanding of how communities in SA and Ghana interact with their food supplies and have led to identification of specific opportunities to improve food supply policies, in ways that create incentives for the production and consumption of healthy, relative to unhealthy foods.

About 40 % of the population of sub-Saharan Africa (SSA) now live in cities, a percentage that is forecast to rise. Two countries already facing higher rates of urbanisation are South Africa (60 %, Statista South Africa, 2023) and Ghana (50 %, Statista Ghana 2023). At present, the urbanisation rates in South Africa and Ghana are 67·8 % and 58·7 %, respectively^(1,2)^. At present, over 70 % of city residents in these countries live in townships or slums – working-class sprawls of formal and informal slum-type housing on the fringes of these urban settlements.

Urbanisation is contributing to changed livelihoods and diets in both rural and urban areas^(3,4)^. Poverty levels pre- and post-COVID have remained steady or increased marginally in some areas, while for others poverty rates have risen rapidly, especially during and post-COVID^(5)^. In addition, rural areas and food systems are being transformed through the penetration of formal retail outlets, often affiliated with international trade and globalisation. Steadily, about half of food imports have composed of cheap ultra-processed foods which are linked to positive changes in weight^(6,7)^. In South Africa and Ghana, the share of the workforce engaged in farming is declining, and rural households are diversifying livelihoods^(8)^. Research in Southern Africa indicates about half of the emerging middle class is rural, and in both urban and rural areas the contribution of purchased and processed foods to overall food expenditure is substantial^(9)^.

Alongside this rapid urbanisation, modernisation and growing food insecurity has been a ‘nutrition transition’^(10)^. Food insecurity trends in South Africa have consistently declined from 29·3 % in 2002 to 11·1 % in 2019 (pre-COVID) but increased post-COVID – however, the current rates (12·2 %) are still lower than in 2002^(11)^. In Ghana, there has also been a decline in food insecurity from approximately 13·6 % in the early 2000s to 4·1 % in recent times^(12)^. South Africa and Ghana are on the same pathway albeit with Ghana on a delayed or slower trajectory. Nutrition transition has created a double burden of malnutrition, in which persisting high rates of stunting and micronutrient deficiencies are now coupled with a rising prevalence of obesity and chronic non-communicable diseases in South Africa and Ghana and increasingly throughout SSA^(13)^. Women are disproportionately affected both by undernutrition and micronutrient deficiencies that impact on their health and reproductive experiences as well as overweight and obesity which contribute significantly to increasing rates of non-communicable diseases such as diabetes, heart disease and certain female cancers^(14)^. Key dietary items implicated in the dual burden of malnutrition throughout the developing world are highly processed food products^(15)^, which tend to have poorer nutritional content – and are more energy dense and higher in salt, fat and/or sugar – than less processed alternatives^(16)^.

The proximal food environment that consumers interact with has a strong influence on decisions and habit formation regarding food purchase and consumption^(17)^. While research exists that has described and analysed the nutrient content of the diets of South Africans and Ghanaians living in poor socio-economic situations^(18)^, there is a paucity of information about how consumers interact with a changing food environment – and in particular, the relative proportion of food acquired from formal (supermarkets and fast food chains) and informal (spazas and food vendors) outlets, or the relative proportions of unprocessed foods or minimally processed foods, processed culinary ingredients, processed foods and ultra-processed foods^(19)^

The sources of food have changed dramatically^(20)^. Corporate supermarket enterprises are now increasingly dominating food markets – in the case of South Africa, five retail supermarket chains collectively account for between 80 % and 90 % of the formal food retail market in South Africa^(21)^. Although currently only 30 % of food is purchased in the formal sector in Ghana, foreign retailers view this as a growing market^(10)^.

Production and flows of foods in and into South Africa as well as the number and range of companies involved in manufacturing and retail have recently been examined, in the context of food security and nutrition^(14)^. However, there is still little known about the nature of the value chains, industry dynamics and policies driving the proximal food environments in peri-urban settings. Although the food environment has a significant impact on both undernutrition and Non Communicable Disease (NCD) risk, the diets of urbanisation and modernising populations in Africa and how they make decisions about food acquisition remain largely unknown. Even more importantly, little is known about the ways in which members of poor and marginalised populations engage with these food environments, and how factors such as income, energy poverty, transport needs, ideological and cultural preferences, gender, perceptions of food quality, nutritional value and status interact to shape consumer choice. Understanding these dynamics between consumers, food environments and food systems can provide insights to inform effective interventions that both support consumers to make healthier food choices and create incentives for healthier food within the food environment. Policies that govern food environments and broader food systems create incentives that influence the price, accessibility and acceptability of healthier foods. These include decisions made by ministries of finance, trade, industry/investment, and agriculture, which interact with food industry dynamics and political considerations regarding food supply and demand. There are many possible policy interventions that can be instituted at municipal, provincial and national government level to regulate and improve the food environment^(15)^. Some of the effective policies suggested by the World Health Organisation include restricting the advertisement of unhealthy foods and fiscal policies like taxation to reduce the consumption of Sugar Sweetened Beverages (SSB)^(22)^. Yet, with a few notable exceptions (salt regulation and tax on sugary beverages in South Africa; food standards regarding fatty meat in Ghana), very few such policies have been implemented in African countries^(16)^. There is a need both to identify the key policy levers that exist at the level of food systems and value chains to shape the nature of local food environments – and to identify how the agency of poor and vulnerable populations themselves can be effectively engaged in these spaces.

The ROFE study sought to better understand the changing nature of food consumption and value chains and their drivers in SSA within the context of nutrition transition in Africa in order to identify opportunities to align policies across sectors to promote healthy diets and prevent NCD.

## Methodology and approach

The ROFE study was implemented in South Africa and Ghana and was led by the Department of Science and Technology/National Research Foundation Centre of Excellence in Food Security, based at the University of the Western Cape. The research in Ghana was supported by Kwame Nkrumah University of Science and Technology, Kumasi, Ghana. Through collaboration with researchers at the Universities of Sydney (Australia), São Paulo (Brazil) and Montreal (Canada), both African institutions’ research capacity was enhanced.

The study was implemented in three phases. Phase 1 determined household food consumption decision-making and related factors. Phase 2 assessed the value chains of healthy and obesogenic food commodities, while Phase 3 utilised a political economy analysis approach to elicit information to inform development of stronger policies to promote nutrition and support other food system objectives. The learnings from this project were to inform the design of food system policies throughout the region.

We purposefully identified four study localities, two in Ghana and two in South Africa. In South Africa, Khayelitsha (peri-urban) and Mt Frere (rural Eastern Cape), and in Ghana Ejuratia/Ankaase (rural locality in Ashanti Region) and Ahodwo (urban locality in Ashanti Region) were identified. The study sites were familiar to the University of the Western Cape and KNUST and thus minimised possible barriers to access. In identifying the study localities, we considered:

- Sociodemographic status (focus on poor populations).
- Migration pathways related to urbanisation.
- The direction and nature of local food system restructuring and retail penetration.
- Inclusion of a rural and urban/peri-urban locality in each country. In South Africa, there was ongoing work (PURE in Mt Frere and SMART-2D in Khayelitsha) on diet-related NCD^(23)^ that complemented the food system work in this proposal.

Location-based workshops were conducted to bring together researchers with food system regulators, relevant government officials (policymakers, town planners, health system experts, etc.) and representatives of formal and informal food value chains. Together, they worked through the collected evidence and proposed policy responses to promote more equitable and gender-sensitive access to healthy food-related livelihoods. This was conducted in a structured way referred to as ‘learning journeys’ in collaboration with the Southern African Food Lab.

### Summary of Supplement findings

The ROFE study is one of the groundbreaking food environment research projects in SSA. By focusing on two countries from two different subregions within SSA which are relatively progressed in terms of nutrition transition, the ROFE findings provide a good understanding of the extent of the impact of food environment on consumption, characteristics of value chains of healthy and unhealthy foods, as well as the potential for improved governance and policy that is relevant to the region. The supplement provides the opportunity to share the extensive findings of the ROFE project. Nine papers that describe the process and findings of the three phases of the ROFE project have been presented. Some of the papers focus on phases of the ROFE, while others cut across different phases and explore the linkages between the phases. Brief descriptions of key findings of some of the papers in the supplement are provided.

The paper on ‘dietary intake of low-income adults in South Africa: Ultra-processed Food consumption a cause for concern’ showed the growing consumption of UPF among low-income adults in South Africa. Increasing consumption of UPF depicts a nutrition transition and explains the growing incidence of diet-related NCD. The paper on ‘Nova Food Acquisition and consumption by Lived Poverty Index (LPI) among rural and urban households in South Africa and Ghana’ determined the level of consumption of different NOVA food classes and how these are associated with different food supply systems and household lived poverty. The paper demonstrates that in South Africa, deprived households had reduced odds of consuming both unprocessed foods (OR 0·296 *P* < 0·001) and ultra-processed foods (OR 0·358 *P* < 0·001) compared with non-deprived households. In Ghana, deprivation using LPI was not significantly associated with food consumption by NOVA classification. The paper on ‘Characterising household healthy and unhealthy food consumption, retail and distribution in Ghana’ explored how consumption, retail and distribution of ‘obesogenic’ *v*. ‘healthy’ food commodities in Ghana are related to sociodemographic variations and weight status. The strongest driver of obesogenic food availability in the country was found to be importation, whereby most of the unhealthy food commodities had their origins outside Ghana. These findings provide clear reasons why policies that regulate importation of unhealthy foods should be enacted to promote and protect public health.

The ‘Operationalising Multi-sectoral Food- and Nutrition-related Policies to curb the Rise in Obesity in Ghana’ study is grounded in a conceptual framework focused on power, triangulated key-informant interviews, learning journeys and relevant policy documents to examine the governance of the food and nutrition policy space with reference to interests and power among stakeholders. It reports that power relations generated tensions, leading to weak multi-sectoral coordination among actors within the nutrition policy space. Governance and funding issues were identified as reasons for the weak multi-sectoral coordination. It observed that formal power rested with government institutions, while the private sector and Civil Society Organisations (CSO) pushed to be invited during policy formulation. There were no observed structures at the subnational levels for effective links with the national level. The paper on ‘Strengthening the governance of food systems for health in Africa: a political economy analysis of food policy in South Africa and Ghana’ examined underlying political economy factors that enable or impede the integration of nutrition considerations into food system governance. It used a comparative political economy analysis of data collected through (1) value chain analyses of selected healthy and unhealthy commodities and (2) food system policy analyses, using a theoretical framework focused on power, politics, interests and ideas. The findings of this paper show that although nutrition was a stated policy priority in both countries, policy responsibility was located within the health sector, with limited integration of nutrition into food system sectors (including Agriculture, Trade and Industry). Contributing factors included conceptions of policy responsibilities for nutrition and food systems, dominant ideas, and narratives regarding the economic role of the food industry and the purpose of food system policy, the influence of large food industry actors, and limited institutional structures for cross-sectoral engagement and coordination.

## Conclusion

Together, the findings of the ROFE study presented in this supplement have (1) increased understanding of how communities in SA and Ghana interact with their food supplies, and the factors that influence this, and (2) led to identification of specific opportunities to improve food supply policies, in ways that create incentives for the production and consumption of healthy, relative to unhealthy foods.
